# FDA orphan products clinical trial grants: assessment of outcomes and impact on rare disease product development

**DOI:** 10.1186/s13023-020-01514-5

**Published:** 2020-09-03

**Authors:** Kathleen L. Miller, Christine Mueller, Gumei Liu, Katherine I. Miller Needleman, Janet Maynard

**Affiliations:** US Food and Drug Administration, Office of the Commissioner, Office of Orphan Products Development, 10903 New Hampshire Avenue, Silver Spring, MD 20993 USA

**Keywords:** Clinical trials, Grants, Orphan drug act, Orphan product grants program, Rare diseases, Rare disease medical product development, US Food and Drug Administration

## Abstract

**Background:**

The Office of Orphan Products Development (OOPD) of the United States (U.S.) Food and Drug Administration (FDA) has awarded over 700 grants to conduct clinical trials of medicals products for rare diseases since 1983, leading to over 70 marketing approvals. However, despite recent progress in rare disease product development, thousands of rare diseases still have no approved treatments. An assessment of this clinical trial grants program was undertaken to provide an in-depth analysis of the characteristics and outcomes of the program. Results of this analysis will be used to inform future goals of the program, as well as internal data collection to continue to maximize the program’s impact in supporting rare disease product development.

**Results:**

Between fiscal years 2007—2011, OOPD funded 85 clinical trial grants. These grants spanned 18 therapeutic areas, included all pre-approval phases (Phases 1–3), and approximately 75% of the grants studied small molecule drugs. Nine (11%) product approvals, of seven drugs and two devices, were at least partially supported by grants funded within this 5-year timeframe. Four of the seven drugs approved were new molecular entities (NMEs). The average time from funding to approval was seven years. We also found a suggested association between collaboration with multiple types of stakeholders and the success of grants, where we defined success as either positive or negative study findings or a future marketing approval.

**Conclusions:**

The clinical trials funded by OOPD provided valuable information for future product development, and there were a notable number of approvals that occurred using the support of the grants program. There was a suggested association between collaboration and successful outcomes. Efficient and innovative trial designs and collaboration among stakeholders appear vital to continue to effectively bring products to rare disease patients. Ongoing program assessments will ensure that the funding continues to be used to optimally meet the treatment needs of the rare disease community.

## Background

In 1983, Congress passed the Orphan Drug Act (ODA) to provide economic incentives for the development of drugs for rare diseases. The ODA defines a rare disease or condition as one that occurs in fewer than 200,000 people in the U.S. [1] In the European Union, similar legislation was passed in 1999 and defines a rare disease as one that affects fewer than 5 per 10,000 within the European Union [2]. Some of the differences between the legislation include the availability of tax credits in the United States but not in the European Union, and differences in the length of marketing exclusivity [2].

One of the incentives created by the ODA was the funding of clinical trials for rare diseases, a program that is administered within the U.S. Food and Drug Administration (FDA) by the Office of Orphan Products Development (OOPD). This Orphan Product Grants Program supports the clinical development of drugs, biologics, devices, and medical foods for use in rare diseases or conditions where no current therapy exists or where the proposed product will be superior to existing therapy [3]. It provides grants for clinical trials on safety and efficacy that will either result in, or substantially contribute to, marketing approval of medical products for rare diseases. This includes studies in any phase of clinical development and includes studies of new indications for previously approved drugs.

Grants provided by the Orphan Products Grants Program are available to any foreign or domestic, public or private, for-profit or nonprofit entity (including state and local units of government, but not federal agencies). Applications are individually reviewed and scored for scientific and technical merit by an independent ad hoc panel of rare disease and regulatory experts. Applications are funded based on their objective review scores, availability of federal funds, and relevance of the project to program priorities. A unique attribute of the program is the incorporation of input from the relevant FDA review divisions to help determine whether the proposed study will provide acceptable data that could contribute to product approval. Funded grants are assigned Project Officers from within OOPD who oversee study progress and assist if issues arise with the study.

Since the program’s inception, OOPD has funded over 700 studies totaling over $420 million in dispersed funds [4]. These studies have contributed to more than 70 marketing approvals for the treatment of rare diseases. However, with over 7000 rare diseases there remains a clear need for the development of treatments for the majority of rare diseases [5]. Many challenges in rare disease product development have been recognized, such as: the heterogeneity of disease manifestations and progression, little existing knowledge on the presentation and course of most rare diseases, and the limited number of geographically dispersed patients [6]. OOPD also began funding natural history studies in 2017 to facilitate solutions to some of these issues.

The purpose of this research was to assess a historical subset of this program by gathering data on characteristics and metrics of the grants. The results of this review will provide an in-depth look at the features and outcomes of OOPD clinical trial grants, which will help inform future program goals in supporting innovative and efficient trials that support the development of new therapies for rare diseases with an unmet need.

## Methods

All grants funded during a 5-year period, fiscal years 2007—2011, were included in this assessment. This period was chosen for two reasons. First, grants that were funded before this period were not available electronically. Second, more recently awarded grants may not have had enough time elapse to see long-term outcomes, such as marketing approvals.

Data was gathered on the baseline characteristics of the grants, as well as outcomes, as of December 2019. The baseline grant characteristics that were collected included: medical product type, clinical trial phase, number of study sites, whether the product ever received an orphan drug designation, therapeutic area, and financial metrics. Data was also collected on whether a grant had academic, industry or patient group collaboration at the time of the application. Grants that did were categorized as “collaborative”.

These characteristics were obtained from two main sources: the application itself, and two internal OOPD databases (one for the grants program and one for the orphan drug designation program). After the data was collected, it was analyzed for trends to determine the most informative characteristics, and only those characteristics are presented in this paper.

Outcomes were collected from an internal OOPD grants program database. There were two main outcomes of interest: product regulatory approval and study finding. An approval was defined to be if the medical product had been approved by the FDA for the disease that was studied in the grant. The clinical trial funded within the grant did not have to be a pivotal study for the approval.

Study findings were determined using the final report submitted by the principal investigator at the conclusion of the grant, as well as any publications resulting from the grant. OOPD project officers made a determination as to whether the study findings were positive, negative, or equivocal based on definitions of the study aims. A positive study finding was defined as efficacy and/or safety findings that led to favorable conclusions of the study drug in the studied rare disease. A negative study finding was defined as findings on efficacy and/or safety that led to unfavorable conclusions about the study drug in the studied rare disease. An equivocal study finding was defined as inconclusive findings on safety and/or efficacy.

## Results

Between FY 2007—2011, OOPD awarded funding to 85 new grants. Of these, 60 (71%) were to study small molecule drugs, 19 (22%) were for biologics, 5 (6%) were for medical devices, and one (1%) was a medical food.

The majority of the grants were for Phase 2 trials (48, 56%). Phase 1 trials accounted for 18 (21%) of the grants, and Phase 3 accounted for 19 (22%).

Most of the grantee institutions (70, 82%) were nonprofit entities (universities, hospitals, and patient groups). Medical product industry grantees accounted for 18% (15) of grants in the study period.

**Fig. 1:**
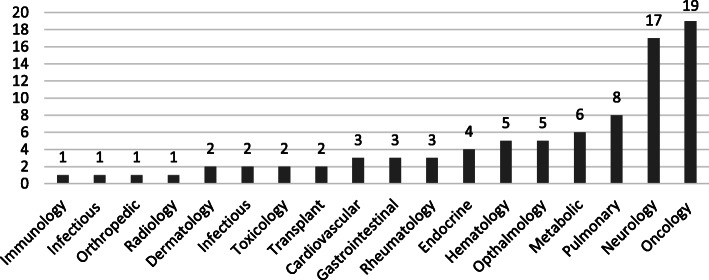
Distribution of Funded Grants by Therapeutic Area, FY 2007—2011 (*N* = 85)

The largest therapeutic area represented was oncology with 19 (22%) grants (Fig. 1). Neurology was the second largest therapeutic area with 17 (20%) grants.

### Study findings

Of the 85 grants, study findings were available for 66 grants. Of the 66 completed grants, 46 (70%) had demonstrated positive study findings, 9 (14%) demonstrated negative study findings, and 11 (17%) demonstrated equivocal study findings.

Study findings were not available for 19 grants because their funding had been terminated early due to various reasons, such as insufficient enrollment or unforeseen circumstances that made continuing the grant infeasible. Therefore, these 19 grants did not have a descriptive study finding available, and meaningful conclusions could not be drawn from them. None of the characteristics of these grants indicated that they were different from completed grants: 79% (15) were small molecule products; 58% (11) were for Phase 2 trials; 79% (15) of the grantee institutions were nonprofit entities; and, more than half were for either oncology or neurology products (five of each).

**Fig. 2:**
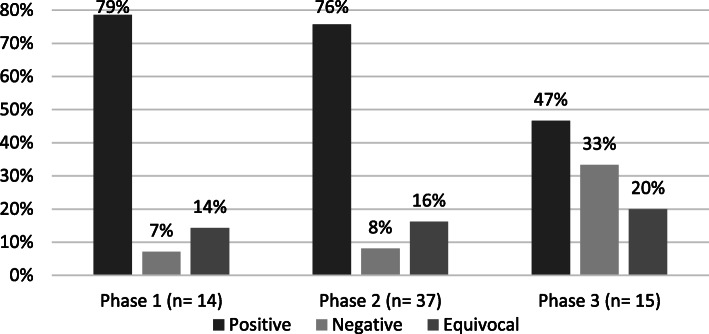
Distribution of Study Findings by Trial Phase, FY 2007—2011 (*N* = 66)

Figure 2 shows the distribution of grants by phase of development that had positive, negative, or equivocal findings. Phase 1 and Phase 2 studies had very similar results by study finding, where more than 75% of the results were positive. Phase 3 studies had much more varied outcomes. For Phase 3 studies, positive findings were much reduced in comparison to Phase 1 and 2 studies (47% for Phase 3 versus greater than 75% for both Phase 1 and 2) and positive findings in Phase 3 studies occurred in a similar proportion to negative outcomes. However, the proportion of equivocal findings for Phase 3 grants was similar to those seen with Phase 1 and Phase 2 grants.

**Fig. 3:**
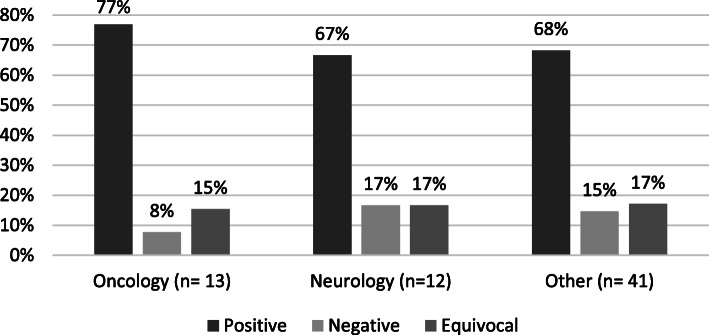
Distribution of Study Findings by Therapeutic Area, FY 2007—2011 (*N* = 66)

Figure 3 shows the distribution of study findings by therapeutic area (oncology, neurology, and other, which includes sixteen other therapeutic areas). The oncology and neurology therapeutic areas were the largest percentage of the 85 awards funded, and thus are shown separately from the other areas. The proportion of equivocal outcomes was almost identical across the three groups. Positive results were proportionally slightly higher for oncology products. The largest difference is that oncology products had, proportionally, about half as many negative outcomes as the other two categories.

**Fig. 4:**
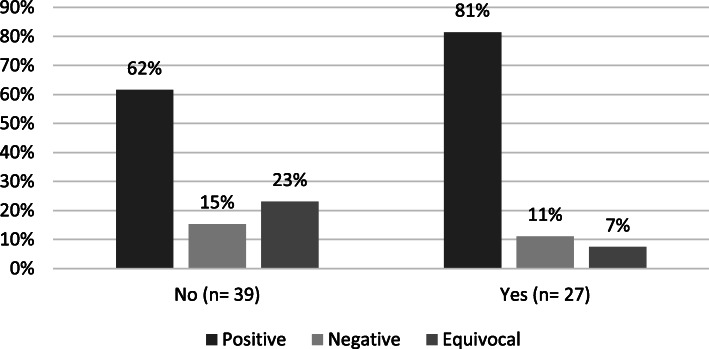
Distribution of Study Findings by Collaboration with Industry or Patient Group, FY 2007—2011 (*N* = 66)

Figure 4 shows the distribution of study findings by studies that had collaboration with an industry or patient group partner at the time of application for funding. The results indicate that the proportion of collaborative grants that had positive findings was 31% higher than for the non-collaborative grants. Additionally, the proportion of equivocal findings was over 200% higher for non-collaborative grants than for those that utilized collaborations.

### Approvals

Out of the 85 grants supported by OOPD during this 5-year period between FY2007–2011, nine (11%) of these grants supported nine product approvals (Table 1). Specifically, seven drugs and two devices were approved. All seven drugs approved had received an orphan drug designation from OOPD (all but one prior to being funded). Prior to funding, OOPD designated the Berlin Heart EXCOR Pediatric Ventricular Assist Device as a humanitarian use device.

**Table 1:** Characteristics of Funded Grants in FY 2007—2011 that Led to FDA Approvals, (*N* = 9)

Year Approved (First Funded)	Generic (Trade Name)	Disease (Therapeutic Area)	Study Phase at Funding	PI Institution	Single or Multiple Study Sites	Non-Academic Collaboration at Application
2012 (2007)	Ivacaftor (Kalydeco)	Cystic Fibrosis Subjects with G551D (Pulmonary)	Phase 2	Industry	Multiple- 8 sites	Patient group
2013 (2008)	Topical nitrogen mustard, Meclorethamine (Valchlor)	Mycosis Fungoides (Oncology/Hematology)	Phase 2	Academic	Multiple- 2 sites	Industry
2015 (2011)	Asfotase alfa (Strensiq)	Hypophosphatasia (Endocrinology)	Phase 2	Industry	Multiple- 7 sites	None
2015 (2008)	Parthyroid Hormone (Natpara)	Hypoparathyroidism (Endocrinology)	Phase 3	Academic	Single	Industry
2015 (2007)	Sirolimus (Rapamune)	Lymphangioleiomyomatosis (Pulmonary)	Phase 3	Academic	Multiple- 8 sites	Patient group & Industry
2016 (2011)	Cheatham Platinum Stent System	Aortic Wall Injury Associated with Aortic Coarctation (Cardiovascular)	Phase 3	Academic	Multiple- 19 sites	Industry
2017 (2009)	Berlin Heart EXCOR Pediatric Ventricular Assist Device	Bridge-to-Heart Transplantation in Children (Cardiovascular)	Phase 2	Academic	Multiple- 13 sites	None
2018 (2008)	Fish Oil Triglycerides (Omegaven)	Reversal of Parenteral Nutrition-Associated Cholestasis (Gastrointestinal)	Phase 2	Academic	Single	None
2019 (2008)	Tafamidis meglumine/ /Tafamidis free acid (Vyndaqel/Vyndamax)	Familial Amyloid Polyneuropathy (Neurology)	Phase 3	Industry	Multiple- 8 sites	None

The average time from funding of the study to marketing approval for these products was 7 years. Four of the seven drugs approved were new molecular entities (NMEs), meaning that the active ingredient had not been previously approved by the FDA. The other three drugs were approved for new indications of drugs that had been previously approved for another indication.

The product approvals spanned a range of therapeutic areas. There were two approvals within the cardiovascular, endocrinology, and pulmonary therapeutic areas, and one approval within the gastrointestinal, neurology, and oncology therapeutic areas.

To date, no Phase 1 studies funded during this period have supported a marketing approval. Five of the nine studies (56%) that supported marketing approvals were Phase 2 clinical trials. The majority of the Principal Investigators (PIs) of the grants were based at an academic institution (6, 67%). All but one of the grants that led to an approval involved a collaboration between the PI institution and other academia (i.e., multiple study sites), industry, or a patient group at the time of the application for funding.

### Discussion

This 5-year assessment illuminated several characteristics and outcomes of interest for the grants funded in this range. Interestingly, 11% of funded grants supported a marketing approval, for nine different medical products, for the treatment of rare diseases. We consider this to be a very successful outcome, given the structure of this grants program, and the average rate of success for drug development generally [7–9].

Also remarkable was that the average time from initial funding of these grants to approval was 7 years, as it is estimated that it can take anywhere more than 15 years to complete all three phases of clinical development and receive marketing approval [10]. We believe this supports the hypothesis that access to this unique funding mechanism translates to well-designed rare disease studies, allowing these studies to continue development, and potentially piquing the interest of other investors to continue supporting the development programs.

This assessment also found several interesting relationships between the characteristics of funded grants and the outcomes of those grants. We found that a wide breadth of therapeutic areas was represented in the funded grants. This result is not completely surprising, given that the grants were open to all rare diseases and were funded based on scientific merit. However, demonstrating that grants were not limited to one or two therapeutic areas shows OOPD’s commitment to facilitate drug development for all rare diseases.

A central finding of this assessment was that while the Phase 1 and 2 trials were more likely than the Phase 3 trials to have positive study findings, only the Phase 2 and 3 trials resulted in marketing approvals. However, there may be some confounding issues here, as Phase 3 trials may have more stringent endpoints, leading to a high bar for success, and therefore it may be more likely that Phase 3 trials have more negative outcomes [11].

These results highlight the challenges in selecting the optimal phases of clinical trials to fund in this grants program. While funding more trials in Phase 1 or 2 may lead to more information (positive or negative) being accrued for the product in the disease being studied, funding more Phase 2 and 3 trials may lead to more product approvals. Both results are important and provide value to patients and clinicians, therefore it is difficult to determine whether one particular phase should be prioritized over another. With these results in mind, OOPD has determined that it is important to continue to fund all phases of clinical trials based on the scientific merit of the study and whether the study design will allow for the most benefit to patients and rare disease research.

However, it must be noted that OOPD funding in early stages of development (Phase 1 or 2) may be the most needed. Private sources of funding (such as from venture capital or licensing/development agreements with pharmaceutical companies) may be limited for products in the earliest phases of development, when the programs at their most “risky” due to the number of unknown factors present [12]. These private sources may be more available at later stages of development, when the product has been “de-risked” by acquiring more knowledge of safety and efficacy [13]. Public sources of funding, such as the OOPD grants program, may play an important role in de-risking these products at the earliest clinical development stages. While OOPD does not plan on prioritizing the funding of Phase 1 or 2 trials, using the funding as a de-risking mechanism (whether through obtaining greater knowledge of the product by completion of the trial or by using the OOPD funding as seed capital) is an important goal of the grants program.

It is also important to note that having early input and interaction with the FDA review divisions, for all study phases, may facilitate better designed and efficient studies that have a greater chance of success. Encouraging efficient and innovative trials through all phases of development, such as through adaptive and seamless trial designs, basket and umbrella trials studying multiple products or rare diseases, and data modeling and simulations, may also lead to more efficient product approvals [14].

Another main finding of this assessment was that oncology products had, proportionally, about half as many negative outcomes as the other two therapeutic areas. It is possible that this is simply due to the fact that, when compared to other diseases, some cancers may be more scientifically understood in terms of mechanism of action, disease progression and potential targets, leading to better defined endpoints [15]. There also may be more established infrastructure in place, across multiple study sites, for cancer research. These advances could lead to more definitive trial endpoints, more effective study designs, and simpler patient recruitment, which in turn could lead to the lower negative and equivocal study outcomes [16]. Regardless of the differences between diseases, OOPD remains committed to its mandated mission of funding clinical trials for all rare diseases and helping less understood diseases also have access to important funds to support product development.

The last main finding of this assessment was the importance that collaborations played in successful outcomes. First, for study findings, the proportion of non-collaborative grants that had equivocal results was over 200% higher than for collaborative grants. A study finding of positive or negative is important because those findings are ‘actionable’; they suggest that a medical product should either continue being developed for that disease, or that the product does not work in that disease, and therefore development should end, potentially even giving rise to other targets that may be pursued. Having an equivocal outcome means that the study may have to be repeated in order to find an actionable outcome, which increases both the time and money needed to answer the research question. Decreasing the number of equivocal study findings is therefore important to both grantees and funders. Encouraging collaborations may help to generate more actionable findings.

Second, 89% of the approvals in this study had a collaboration in place at the time of the grant application. This provides even greater evidence of the importance of collaboration in having successful outcomes. However, these percentages likely underestimate the true number of collaborations. This assessment only included collaborations that were in place at the time of application, and therefore would not capture collaborations that were initiated after the application was submitted.

There are many pathways by which a collaboration could facilitate an academic grantee having a better chance for a successful outcome [17, 18]. A collaboration with a patient group could help with increasing enrollment in the study, knowing what outcomes are most important to patients, increasing patient input into the trial, or even gaining additional study funding [19]. A collaboration with an industry sponsor could facilitate procurement of the study product (if the collaborating company is manufacturing it), provide relevant proprietary information about the study product, and allow for additional funding, regulatory support and knowledge to move forward with product development [20]. All of these pieces play an important role in medical product development, and OOPD intends to encourage future grantees to form collaborations between necessary stakeholders and include patient input throughout product development.

The results of this assessment will be used to inform the goals of future OOPD clinical trials funding opportunities. In the near-term, the results have led to OOPD focusing the program on diseases with unmet medical needs (regardless of therapeutic area), grants for early phase studies with efficient and innovative study designs (to better utilize FDA regulatory knowledge), and grants that include the use of existing infrastructure and significant collaborations between stakeholders. In the long-term, the results suggest that OOPD should continue to assess the goals of the program by tracking the long-term outcomes of grants to increase the impact of the program and provide models for more efficient development of products for rare diseases.

### Limitations

The main limitation of this assessment is that any differences we find between funded grants can only be interpreted as suggested associations, not causation, as no statistical analysis was performed. We cannot be sure exactly which characteristics of the grants led to the outcomes. However, because the results of this study will not limit the grants program (but rather encourage additional dimensions on which grantees can compete), not having a causal analysis should not have a negative impact on the assessment.

A second limitation of the study is that it provides only a snapshot into the grants program. The studied grants were funded almost a decade ago and may not reflect the characteristics of current grants or the current research landscape. It also may not reflect changes in the larger rare disease medical product development space, such as the rise of the development of biologics, an increased understanding of the pathophysiology of rare diseases, increased focus by groups on particular therapeutic areas over time, use of natural history studies to better define the course of a disease and determine endpoints, and increases in the funding amounts for grants in recent years.

Lastly, there is a limitation that applies to our analysis of study findings. Because the study findings were a determination made using final reports and publications of the grantees, these findings may differ based on the study funded. We believe that, even with this subjectivity, the results of this outcome remain interpretable, of interest, and represent the outcomes presented.

## Conclusion

The purpose of this historical analysis of the OOPD Grants Program was to assess the characteristics and outcomes of funded clinical trials grants and use the results to inform the current and future directions and assessments of the program. We found that the grants program has been successful in generating important clinical information on various rare diseases and supporting many product approvals for important indications. We also found an important suggested association between collaboration and successful outcomes. Efficient and innovative well-designed trials, along with strong infrastructure of the study, including important collaborations among various stakeholders, is necessary to efficiently and effectively bring new treatment options to patients with rare diseases.

## Data Availability

The datasets generated and analyzed during the current study are not publicly available as they contain confidential commercial information. Limited information may be available from the corresponding author upon request but will be reviewed to protect proprietary information.

## Abbreviations

FDA: US food and drug administration
NME: New molecular entity
ODA: Orphan drug act
OOPD: Office of orphan products development
PI: Principal investigator
